# 抑制Src酪氨酸激酶活性对人肺癌A549/DDP细胞耐药性及MDR1和LRP表达的影响

**DOI:** 10.3779/j.issn.1009-3419.2012.09.01

**Published:** 2012-09-20

**Authors:** 洁 律, 玉峰 田

**Affiliations:** 276826 日照，山东省日照市人民医院检验科 Department of Laboratory Medicine, Rizhao People's Hospital, Rizhao 276826, China

**Keywords:** Src酪氨酸激酶, A549/DDP, 多药耐药, MDR1, LRP, Src tyrosine kinase, A549/DDP, Multi-drug resistance, MDR1, LRP

## Abstract

**背景与目的:**

本研究旨在探讨抑制Src酪氨酸激酶活性对人肺癌A549/DDP细胞耐药性及多药耐药蛋白（muti-drug resistance 1, MDR1）和肺耐药相关蛋白（lung resistance-related protein, LRP）表达的影响。

**方法:**

以Src酪氨酸激酶抑制剂作用于A549/DDP细胞，应用Western blot法检测肿瘤细胞Src酪氨酸激活性的变化，CellTiter-Glo发光法检测肿瘤细胞药物敏感性的变化，流式细胞仪检测肿瘤细胞Rh-123含量变化，Western blot法和RT-PCR检测肿瘤细胞MDR1和LRP表达变化。

**结果:**

Src酪氨酸激酶抑制剂可下调A549/DDP细胞中Src酪氨酸激活性，2.5 μM和10 μM Src酪氨酸激酶抑制剂作用肿瘤细胞后，肿瘤细胞药物敏感性提高，逆转倍数（reversal fold, RF）分别为1.59倍和2.10倍，肿瘤细胞中Rh-123含量分别提高了1.21倍和1.59倍，MDR1 mRNA表达分别是对照组的53.8%和27.5%，LRP mRNA表达分别是对照组的59.3%和21.4%，MDR1和LRP蛋白表达水平明显下降。

**结论:**

抑制A549/DDP细胞中Src酪氨酸激酶活性可逆转肿瘤细胞多药耐药性，提高肿瘤细胞药物敏感性，其机制可能与降低细胞MDR1和LRP表达有关。

## Abstract (en)

**Background and objective:**

The aim of this study is to investigate the effect of Src tyrosine kinase inhibition on the drug-resistance as well as the expression of multidrug resistance 1 (MDR1) and lung resistance-related protein (LRP) of the human cis-platinum-resistant lung cancer cell line A549/DDP.

**Methods:**

4-Anilinoquirazoline was used to inhibit Src tyrosine kinase activity in A549/DDP. Western blot analysis was used to detect the Src tyrosine kinase activity. CellTiter-Glo assay was used to detect the drug sensitivity of tumor cells. Flow cytometry was used to detect the intracellular Rh-123 content. Western blot and real-time PCR assay were used to detect the expression of tumor MDR1 and LRP.

**Results:**

4-Anilinoquirazoline can down-regulate the cellular Src tyrosine kinase activity in A549/DDP. After treatment with 2.5 μM and 10 μM of 4-anilinoquirazoline, the cells became more sensitive to the drug and the reversal folds (RFs) of tumor cell sensitivity to the drug were 1.59- and 2.10-fold, respectively. The intracellular content of Rh-123 improved by 1.21- and 1.59-fold, respectively. The mRNA levels of MDR1 were 53.8% and 27.5% of the control, respectively. The mRNA level of LRP was 59.3% and 21.4% of the control, respectively. The expression of MDR1 and LRP protein significantly decreased.

**Conclusion:**

The inhibition of Src tyrosine kinase activity in A549/DDP cells can reverse multi-drug resistance and increase the sensitivity of the cells to the drug. The mechanism may be related to the down-regulation of cellular MDR1 and LRP.

肿瘤细胞产生耐药性是临床肺癌化疗治疗失败的主要原因，克服肿瘤细胞多药耐药是癌症治疗中亟需解决的问题。肿瘤细胞产生耐药性是一个复杂的过程，导致药物在细胞内的蓄积减少或药物靶部位的浓度降低，细胞抗凋亡能力的增强^[1]^。耐药基因的激活与表达增加已被证实与肿瘤的耐药性密切相关，其中多药耐药蛋白（multidrug resistance 1, MDR1）和肺耐药相关蛋白（lung resistance-related protein, LRP）是目前研究较多的耐药机制，二者的表达与肿瘤细胞内或药物靶部位药物浓度有密切的关系。研究^[2]^证明，直接或间接地抑制MDR1和LRP的活性或表达可增加细胞内药物的浓度从而逆转肿瘤的耐药性。

Src是一种分子量为60 kDa的酪氨酸激酶，是细胞蛋白酪氨酸激酶（protein tyrosine kinases, PTKs）中的膜相关Src家族激酶（Src family kinases, SFKs）成员，是最有代表性的非受体酪氨酸激酶之一^[3]^，在调控细胞的增殖、迁移和信号转导等方面均发挥了重要作用^[4]^。大量研究^[5]^表明，Src酪氨酸激酶在多种肿瘤组织或细胞中呈现异常活化的现象，已经被证明与肿瘤细胞的生长、转移、血管新生密切相关，是一个潜在的抗肿瘤药物靶点。最近，Src酪氨酸激酶因被发现与肿瘤多药耐药密切相关而备受关注，研究^[6]^表明抑制Src酪氨酸激酶活性可逆转肿瘤细胞的多药耐药性，但是其机制还不完全清楚，正在引起人们的广泛关注，值得深入研究。

## 1 对象与方法

### 1.1 主要试剂与仪器

人肺癌顺铂耐药细胞A549/DDP购于中国科学院上海生命科学研究院生物化学与细胞生物学研究所，Src酪氨酸激酶抑制剂4-苯胺喹唑啉购自AstraZeneca公司，磷酸化Src单抗、MDR1和LRP单抗购自SantaCruz，Rh-123和CellTiter-Glo试剂盒购自Promega，逆转录试剂盒购自TaKaRa公司，流式细胞仪购自BD公司。

### 1.2 实验方法

#### 1.2.1 细胞与细胞培养

人肺癌顺铂耐药细胞A549/DDP于5%CO_2_、37 ℃、饱和湿度的培养箱中培养传代，以RPMI-1640培养基（含10%小牛血清）培养，0.25%胰酶-EDTA消化传代，所有实验均采用对数生长期细胞。

#### 1.2.2 CellTiter-Glo发光法检测Src酪氨酸激酶抑制剂对A549/DDP增殖的影响

取对数生长期的A549/DDP细胞，以5×10^4^/mL接种到96孔微孔板中，每孔100 μL，37 ℃、5%CO_2_饱和湿度培养过夜使细胞贴壁。对应试验孔加入浓度为0 μM、2.5 μM、10 μM、20 μM、50 μM、100 μM 4-苯胺喹唑啉，继续培养72 h后，按照CellTiter-Glo试剂盒说明书检测细胞活性，即将细胞裂解后，转至遮光96孔板，加入ATP诱导化学反应试剂，混匀，10 min后用酶标仪检测发光值。

#### 1.2.3 CellTiter-Glo发光法检测Src酪氨酸激酶抑制剂对A549/DDP细胞毒作用的影响

取对数生长期的A549/DDP细胞，以5×10^4^/mL接种到96孔微孔板中，每孔100 μL，37 ℃、5%CO_2_饱和湿度培养过夜使细胞贴壁。对应试验孔加入浓度为0 μM、2.5 μM、10 μM、20 μM、50 μM、100 μM顺铂，在此基础上加入0 μM、2.5 μM或10 μM 4-苯胺喹唑啉，继续培养72 h后，按照CellTiter-Glo试剂盒说明书检测细胞活性。

#### 1.2.4 流式细胞术检测细胞内Rh-123浓度的变化

取对数生长期的A549/DDP细胞，加入2.5 μM或10 μM 4-苯胺喹唑啉，不加入4-苯胺喹唑啉做对照，继续培养24 h，加入10 μM Rh-123染液（10μL）继续培养1 h。收集细胞，应用流式细胞仪检测细胞中Rh-123的平均荧光强度，考察细胞中Rh-123的含量。

#### 1.2.5 RT-PCR检测MDR1和LRP的表达水平

取对数生长期的细胞，加入2.5 μM或10 μM Src酪氨酸激酶抑制剂4-苯胺喹唑啉，不加入4-苯胺喹唑啉做对照，培养24 h后，收集细胞，用Trizol法提取各组总RNA，用RT-PCR试剂盒进行逆转录得到cDNA，MDR1上游引物序列：5'-AAAAAGATCAACTCGTACCACTC-3'，下游引物序列：5'-GCACAAAATACACCAACAA-3'；LRP上游引物序列：5′-AGTCAGAAGCCGAGAAAG-3′，下游引物序列：5′-CCCAGCCACAGCAAGGT-3′；以β-actin为内参，上游引物序列：5'-TCCTGTGGCATCCACGAAACT-3'，下游引物序列：5'-GAAGCATTTGCGGTGGACGAT-3'。94 ℃变性3 min后，按下述条件扩增40个循环：95 ℃ 5 s，65 ℃ 35 s，72 ℃ 60 s，循环后72 ℃延伸5 min。

#### 1.2.6 Western blot检测MDR1和LRP的表达水平

取对数生长期的A549/DDP细胞，加入2.5 μM或10 μM 4-苯胺喹唑啉，不加入4-苯胺喹唑啉做对照，继续培养24 h后，收集细胞裂解提取蛋白。BCA法测定细胞裂解物的蛋白含量，取等量蛋白质用12% SDS-PAGE法进行分离，然后转移到PVDF膜上，分别用MDR1抗体（1:800）和LRP抗体（1:500）孵育，4 ℃过夜，以特异性的辣根过氧化物酶连接的二抗温育1 h，洗涤，显示免疫反应条带，以β-actin作为内参（Sigma，1:2, 000）。

### 1.3 统计学方法

使用SPSS 11.5软件进行分析，实验数据以Mean±SD表示。采用单因素方差分析（*One-way ANOVA*）进行比较，以*P* < 0.05为差异具有统计学意义。

## 2 结果

### 2.1 Src酪氨酸激酶抑制剂对A549/DDP细胞增殖的影响

CellTiter-Glo实验结果显示，4-苯胺喹唑啉对A549/DDP细胞增殖的抑制作用呈剂量依赖性，通过细胞抑制曲线得出4-苯胺喹唑啉在2.5 μM时对A549/DDP细胞无明显毒性（抑制率 < 5%），在10 μM时有微弱毒性（抑制率10%-15%）（图 1），因此本研究选择2.5 μM及10 μM 4-苯胺喹唑啉进行研究。

**1:**
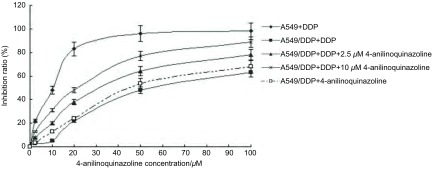
Src酪氨酸激酶抑制剂4-苯胺喹唑啉对A549/DDP细胞增殖的影响和对药物敏感性的逆转作用 The effect of 4-anilinoquinazoline on A549/DDP cells proliferation and reversal effect of drug sensitivity

### 2.2 抑制Src酪氨酸激酶活性可提高A549/DDP对顺铂的敏感性

CellTiter-Glo实验结果显示，2.5 μM和10 μM的4-苯胺喹唑啉可降低顺铂抑制A549/DDP的IC_50_值，顺铂抑制A549的IC_50_值为12.64 μM，A549/DDP的IC_50_值为52.61 μM，而2.5 μM 4-苯胺喹唑啉作用后顺铂对A549/DDP的IC_50_值为33.15 μM，10 μM 4-苯胺喹唑啉作用后顺铂对A549/DDP的IC_50_值为24.98 μM，逆转倍数(reversal fold, RF)分别为1.59倍和2.10倍（图 1）。

### 2.3 抑制Src酪氨酸激酶活性可提高A549/DDP细胞中Rh-123的含量

流式细胞术检测结果显示，Src酪氨酸激酶抑制剂处理后A549/DDP细胞中的Rh-123含量明显提高，2.5 μM和10 μM 4-苯胺喹唑啉处理的肿瘤细胞中Rh-123的平均荧光强度分别比对照组细胞提高了1.21倍和1.59倍（图 2），证明抑制Src酪氨酸激酶活性可降低肿瘤药物外排，提高肿瘤细胞中药物含量，从而提高肿瘤细胞药物敏感性。

**2:**
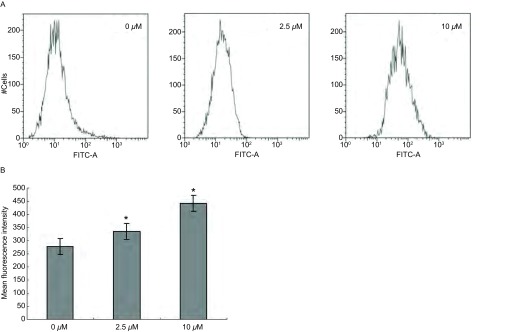
抑制Src酪氨酸激酶活性对A549/DDP细胞内Rh-123蓄积的影响 The effect of Src tyrosine kinase activity on the intracellular accumulation of rhodamine-123 in A549/DDP cells. A: The figure of the effect of Src tyrosine kinase activity on the mean fluorescence intensity of rhodamine-123 in A549/DDP cells; B: A graph representing the analysis of intracellular rhodamine-123 mean fluorescence intensity. Data presented are Mean±SD values from at least three independent experiments. Bars indicate SD.^*^, compared to control group, *P* < 0.05.

### 2.4 抑制Src酪氨酸激酶活性可下调*MDR1*和*LRP*基因转录

RT-PCR结果显示，2.5 μM和10 μM 4-苯胺喹唑啉处理后，A549/DDP细胞的*MDR1*基因的mRNA水平分别为对照组的53.8%和27.5%，*LRP*基因的mRNA表达分别是对照组的59.3%和21.4%（图 3），证明抑制Src酪氨酸激酶活性可下调肿瘤细胞*MDR1*和*LRP*基因的转录，从而降低上述蛋白表达，提高肿瘤细胞药物敏感性。

**3:**
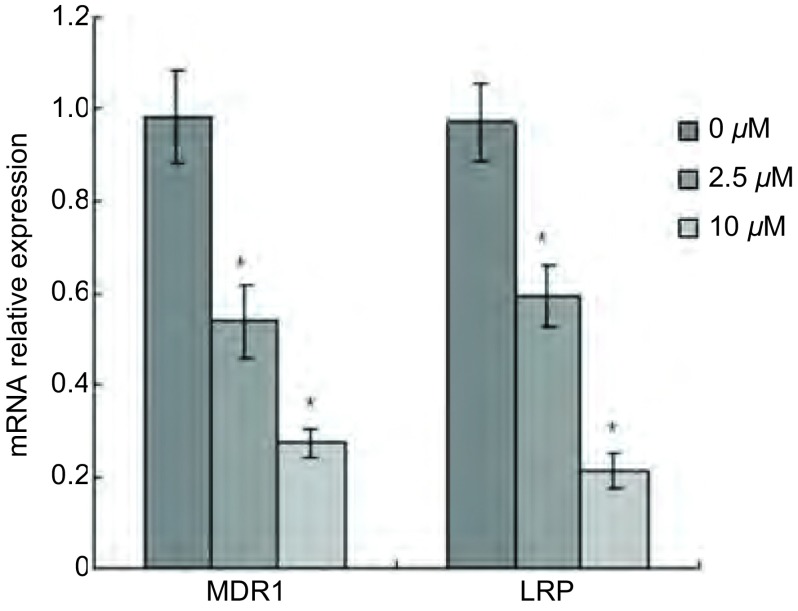
抑制Src酪氨酸激酶活性对A549/DDP细胞MDR1和LRP mRNA表达的影响。 The effect of Src tyrosine kinase activity inhibition on the mRNA expression of MDR1 and LRP in A549/DDP cells. Data presented are Mean±SD values from at least three independent experiments. Bars indicate SD.^*^, compared to control group, *P* < 0.05. MDR1: muti-drug resistance 1; LRP: lung resistance-related protein.

### 2.5 抑制Src酪氨酸激酶活性可下调MDR1和LRP蛋白表达

Western blot检测显示，4-苯胺喹唑啉可降低A549/DDP细胞MDR1和LRP的蛋白水平，且呈现剂量依赖性（图 4），证明抑制Src酪氨酸激酶活性可下调肿瘤细胞MDR1和LRP蛋白表达，从而提高肿瘤细胞药物敏感性。

**4:**
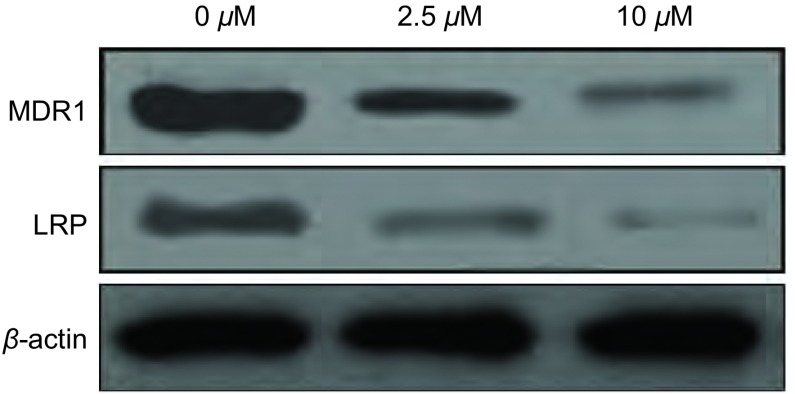
抑制Src酪氨酸激酶活性对A549/DDP细胞MDR1和LRP蛋白表达的影响 The effect of Src tyrosine kinase activity inhibition on the protein expression of MDR1 and LRP in A549/DDP cells

## 3 讨论

肿瘤细胞产生多药耐药性是临床应用化疗药物治疗肿瘤失败的主要原因，研究肿瘤产生多药耐药性的机制及解决方法对提高化疗药物的临床有效性具有重要意义。研究^[7]^显示，肿瘤细胞可通过多种机制产生耐药性：过表达ATP结合盒蛋白超家族（ATP-binding cassette protein super family）等跨膜蛋白基因，将进入肿瘤细胞内的药物被泵出，减少细胞内的药物浓度；过表达具有解毒和DNA修复功能的酶，使细胞毒药物效果减低；下调药物靶点分子表达，降低药物敏感性；激活抑癌基因，抑制药物所致的细胞凋亡等。其中，被广泛研究的机制是多药耐药蛋白MDR1及肺耐药相关蛋白LRP高表达所引起的耐药性，这些蛋白的表达降低肿瘤细胞内或者药物靶附近的药物浓度，导致给予的药物无法有效地到达治疗部位，从而产生耐药性^[8]^。

PI3K-AKT通路是细胞内重要的信号转导通路，在细胞的增殖和生存中起着重要作用，在不同类型的肿瘤中均可见此信号通路的过度表达^[9]^，已有研究^[10]^表明PI3K/Akt路径的活化是肿瘤细胞对药物耐受的关键调控因素。Src酪氨酸激酶活化后，可激活下游PI3K-AKT信号通路，活化下游相关的信号转导通路，通过磷酸化IKKα/β蛋白，提高NF-κB活性，促进*MDR1*和*LRP*基因的转录。

MDR1是能量依赖性跨膜糖蛋白，在肺癌组织中广泛表达，在正常肺组织中低水平表达或不表达。MDR1蛋白具有药泵功能，当肿瘤细胞膜上过度表达MDR1蛋白时，MDR1可结合抗肿瘤药物，通过ATP水解后释放的能量，主动将进入细胞内的药物泵出细胞外，降低细胞内药物的浓度，产生耐药^[11]^。

*LRP*近年来被发现是重要的耐药基因，在肺癌中首先发现，是一种穹隆体主蛋白，在多种耐药细胞株中均有过度表达。LRP可参与组成核孔复合物（nuclear pore complex, NPC）参与细胞核与细胞质间物质交换以及细胞质中的囊泡运输。LRP比MDR1更早地表达在恶性肿瘤中，给予低浓度化疗药后即可见到LRP表达增加，而不需要高浓度化疗药刺激，其过度表达通过调节囊泡和核质的药物转运将药物储存于囊泡，减少其在核与胞质间的比例，可影响药物的胞内转运与分布而导致耐药^[12]^。

由人肺癌细胞系A549连续暴露于顺铂后筛选出的耐药A549/DDP细胞除耐受顺铂外，还对VP-16和CBP交叉耐药，研究^[13]^显示该细胞的多药耐药性与MDR1和LRP的高表达密切相关，是一株良好的工具细胞。Src酪氨酸激酶在肿瘤形成和发展中的重要性已经被人们所熟知，Src的异常活化导致了细胞的癌变并赋予肿瘤细胞存活的能力^[14]^。同时，最近的研究^[15]^显示Src激酶还参与肿瘤细胞抵抗化疗药物，Src酪氨酸激酶抑制剂可以增加肿瘤细胞对化疗药物的敏感性，甚至逆转耐药性。Src酪氨酸激酶与肿瘤耐药性的关系并不十分清楚，Src酪氨酸激酶抑制剂在不同类型肿瘤耐药细胞上的作用值得深入研究。

本研究显示，Src抑制剂4-苯胺喹唑啉可抑制A549/DDP细胞中Src的激酶活性，表现为Src自身磷酸化水平降低，并且CellTiter-Glo试验显示这种抑制作用可进一步转化为提高细胞对顺铂的敏感性。A549/DDP细胞耐药性的主要机制之一是MDR1和LRP过表达，使得细胞外排药物的能力增加导致药物在细胞中的浓度降低，因而其耐药性的逆转很可能与药物在细胞中的蓄积得以恢复有关。Rh-123作为MDR1的底物，可以指示细胞中该蛋白的功能强弱，并指示癌细胞对药物的外排作用，因此我们利用流式细胞术研究了Src抑制剂对细胞Rh-123含量的影响。结果显示，在Src抑制剂处理后，细胞的Rh-123含量明显提高，说明抑制Src酪氨酸激酶活性逆转A549/DDP细胞的耐药性与其抑制药物的外排、提高药物在细胞内的蓄积有关，与我们的推测一致。进一步的Western blot研究显示，抑制Src酪氨酸激酶活性下调了细胞MDR1和LRP蛋白的表达水平，与Rh-123含量实验的结果相符。而real-time PCR研究显示，抑制Src酪氨酸激酶活性下调MDR1和LRP蛋白的表达很可能是通过降低mRNA的转录水平来实现的，其机制可能是抑制Src酪氨酸激酶活性后，PI3K-AKT路径受到阻断，从而下调NF-κB等转录因子活性，降低了MDR1和LRP mRNA的转录水平。

综上所述，本研究显示抑制Src酪氨酸激酶活性可逆转A549/DDP细胞多药耐药性，其机制可能与降低细胞MDR1和LRP的表达，恢复药物在细胞内的蓄积有关。本研究结果为靶向作用Src的药物运用与临床逆转MDR1或LRP介导的肺癌多药耐药提供了实验基础，其具体的作用机制有待进一步的研究。
